# Supplementary material for: Are carbon emissions trading and green financial instruments synergistic? -Comprehensive quantitative research based on content analysis
Source: PLoS One. 2024 Mar 7;19(3):e0298601. doi: 10.1371/journal.pone.0298601 (PMC10919601; doi:10.1371/journal.pone.0298601)
Supplement: S1 Data — (ZIP) [file pone.0298601.s001.zip › Data/地级市Z_F工作报告.zip!/║■─╧/│ñ╔│╩╨/└┤╫╘╣1⁄2╓┌║┼í╛┬φ┐╦╩2╛▌═°í┐_2015.docx]

各位代表：

　　现在，我代表市人民政府向大会报告工作，请连同《长沙市国民经济和社会发展第十三个五年规划纲要（草案）》一并审议，并请各位政协委员和其他列席人员提出意见。

　　一、2015年及“十二五”工作回顾

　　2015年，面对经济下行压力加大的严峻形势，在省委、省政府和市委的坚强领导下，我们按照“稳住、进好、调优”的总要求，抢抓机遇，科学应对，积极作为，完成了市十四届人大三次会议确定的目标任务，实现“十二五”胜利收官。

　　——经济基础更为坚实。实现地区生产总值8510.13亿元（预计数，下同），增长9.9%；规模工业增加值3228.21亿元，增长9.2%；固定资产投资6363.29亿元，增长17.1%；社会消费品零售总额3690.59亿元，增长12.1%；财政总收入1113.48亿元，增长11.01%，其中一般公共预算收入718.95亿元，增长13.61%。经济运行总体平稳。

　　——战略地位更加凸显。湖南湘江新区成为中部首个国家级新区，国家自主创新示范区建设全面启动，两型社会建设纵深推进，长沙在国家总体战略布局中的地位不断提升。

　　——产业发展更具活力。先进制造业加快发展，上海大众等一批重大项目投产；新兴产业迅速崛起，仅长沙高新区就新增58到家等移动互联网企业1083家、总数达1693家；现代服务业快速发展，电子商务交易额、服务外包业务总量分别增长30%、13.2%；现代农业蓬勃发展，新型农业经营主体达1.5万户。

　　——交通体系更趋完善。地铁1号线一期全线轨通，2号线西延线一期试运营，磁浮工程试运行，万家丽路快速化改造竣工通车，湘江新区综合交通枢纽投入运营。

　　——城市管理更富成效。坚决落实省委“五高”要求，城市管理措施和责任落实到街道、社区，市容环境秩序等专项整治成效明显，特别是集中开展大规模拆违控违行动，中心城区违法建筑两年“基本清零”目标一年实现，创造了城市管理新经验。

　　一年来，我们着力抓了七个方面的工作：

　　（一）着力产业转型升级。实施智能制造、新材料等三年行动计划，战略性新兴产业加快发展，新能源汽车产业发展势头强劲，比亚迪电动乘用车项目投产；新材料、电子信息、生物医药等产业产值分别增长10.1%、16.7%、18.3%；高新技术产业增加值达2600亿元，增长18%。依靠技术改造提升产业竞争力，完成技改投资2091.7亿元，方盛制药、博云航空刹车等技改项目成效明显；工程机械产业积极开拓发展领域，加快向智能化、多元化转变；淘汰落后产能，宁乡县率先实现小煤矿全关闭。园区规模以上工业增加值增长10.7%，浏阳经开区晋升千亿园区，长沙经开区获批国家生态工业示范园区，隆平高科技园、金霞经开区获批国家电子商务产业示范基地，宁乡高新区获批国家新型工业化产业示范基地，长沙（国家）广告产业园开园。加快服务业发展，旅游业总收入、文化产业总产值、金融业增加值分别增长13.37%、11.8%、24.2%，现代服务业综合试点继续获评全国先进。

　　（二）着力发展动力转换。坚持创新驱动、项目拉动、开放带动，深入推进大众创业、万众创新。湖南湘江新区高端产业发力，中兴通讯、湘江欢乐城等项目建设加速推进。实施鼓励自主创新“33条”，国家自主创新示范区建设首批54个示范项目全面启动。组建智能制造研究总院、机器人研究院，成立11个院士专家市级工作站。获批全国小微企业创业创新基地城市示范，5个示范性众创空间及未来方舟创业创新基地加快建设，三一众智新城正式启动，长沙县率先建设农村创客平台。成立中部首个知识产权交易中心，城市万人有效发明专利拥有量居省会城市第4位，再次获评国家知识产权示范城市。

重大项目建设成效显著，624个重点项目完成投资2095亿元，蓝思科技蓝宝石、千山大健康产业基地、远大住工、欧智通等项目顺利推进。招商引资亮点纷呈，实际利用外资增长11%，宜家、华强、中国通号等项目签约落户，首届全国异地长沙商会联席会成功举办。新增上市公司5家，新三板挂牌企业40家。举办规模会展193个。国际定期航线达20条。霞凝货运站获批国家一级铁路货运基地。湘赣边区域合作迈出实质性步伐。湖南进出口商品展示交易中心建成运营。全市进出口总额达125亿美元，增长8.1%。

　　（三）着力重点领域改革。9大类60项改革有序推进。新型城镇化、农村宅基地、公共文化服务标准化等19项“国字号”改革试点启动实施。户籍制度、公立医院、不动产登记、大瑶强镇扩权等改革稳妥推进。加快政府职能转变，完成政府机构改革和乡镇区划调整。深化行政审批改革，承接新下放省级管理权限27项，向区县（市）再下放权限39项，彻底取消非行政许可审批事项，编制并公布4417条政府部门责任清单事项，长沙高新区推行负面清单制度。开辟重大项目审批绿色通道，实现投资项目网上审批。深化商事制度改革，率先实施“三证合一、一照一码”，新增商事主体10.7万户，增长8.7%。加强投融资体制改革，设立产业投资、城市发展等基金，实施政府和社会资本合作项目28个。深化财税体制改革，推行全口径预算管理、预决算公开。加快国资国企改革，组建国资、国投、交通三大集团。推进两型社会建设综合配套改革，实施节约集约用地标准化和生态补偿制度，单位地区生产总值综合能耗降低2%以上。完成城区公证、城镇供排水、城市园林绿化、高铁新城管理等体制改革。

　　（四）着力品质城市建设。坚持规划引领，强化文化名城、路网建设、生态景观等专项规划，优化湘江西岸、浏阳河两岸规划设计，启动解放垸、朝正垸规划编制。基础设施不断完善，地下综合管廊建设国家试点有序实施，城际铁路、黄花机场东扩等重大项目进展顺利，岳宁大道、坪塘大道、芙蓉北大道、湘江北路、新开铺南路建设和芙蓉南路提质改造加快推进，洞株公路全线通车，京港澳高速下沉改造、火车货运新北站一期建设全面完工，打通断头路、瓶颈路23条。优化公交线网，填补公交空白，公交都市创建加快推进。依法拆除违法建筑1442万平方米，处置闲置土地1.25万亩，清理“围而未建”土地2.04万亩。完成棚改3.06万户468万平方米，超额完成全年任务，都正街、碧湘街、桐梓坡大板房等棚改项目成效明显。石人村、火炬村等20个城中村改造全面推进。取缔和迁移湘江趸船91艘。谷山体育公园、沙湾公园建成，湘府文化公园建设、烈士公园二期提质加速推进，浏阳河风光带加快建设。家禽批发市场、红星农副产品市场迁建开工，高桥物流市场完成外迁。实施“清霾、碧水、静音、净土”行动，淘汰黄标车2.3万辆，全年空气质量优良天数258天，比上年增加31天；强力实施湘江保护与治理“一号重点工程”，主城区101个排水口实现全面截污。深入实施“三年造绿大行动”，新增造林10万亩、绿地9000亩，拆墙开放绿地208.47万平方米，森林覆盖率达54.9%。启动重点区域和道路亮化绿化，市容市貌更加整洁有序。

　　（五）着力农业农村发展。开展永久基本农田划定，完成建设用地补充耕地17920亩，新增耕地3399亩，“米袋子”、“菜篮子”保障更加有力；农产品直销工程深入推进，建成专业农产品集中配送中心6家，发展直销网点1700多家；实现农产品加工业销售收入1550亿元。成功举办中部国际农博会。宁乡县获评全国电子商务进农村综合示范县。实施农业基础设施和产业项目109个，完成投资31.66亿元。建设水利工程3万余处，除险加固小型水库152座，山塘清淤扩容5.86万亩；新建农村公路617公里，改造农村公路303公里、危桥34座、危房9521户，新建改建无害化户厕1.03万座；长沙县率先实现镇街公交全覆盖。开慧、莲花、沩山等特色镇品质明显提升，湘江古镇群建设有序推进，浔龙河等16个美丽乡村建设取得新进展，浏阳市获批全国休闲农业与乡村旅游示范县（市），灰汤成功创建首批国家级旅游度假区，永安镇、白马桥镇获评全国文明镇。

　　（六）着力社会民生保障。重点民生实事项目全面完成。城乡居民人均可支配收入分别达3.9万元、2.3万元，增长8.5%、8.6%。居民消费价格指数为101.1，物价总体稳定。城镇新增就业14.29万人，失业人员再就业3.73万人，城镇登记失业率控制在3%以内。实现工伤保险职业人群全覆盖，社保“一卡通”统筹推进。城乡低保保障20.3万人次，医疗救助30万人次。建设保障性住房4.86万套，推行公积金异地贷款，完成6.4万户房产办证遗留问题处理。改扩建敬老院12所，新增养老床位3000余张。建成社区服务中心26个，新增社区服务用房1.5万平方米。公立医院全部取消药品加成，市中心医院全科医生培训基地、滨水新城医院等项目正式开建，新改扩建乡镇卫生院21所、社区卫生服务中心8所、村卫生室274所，计生特殊困难家庭扶助有效落实。新改扩建公办幼儿园45所、城区中小学校10所，提质改造薄弱学校115所，教育质量综合评价、特殊教育等国家试点有序推进。“三馆一厅”全面开放，音乐厅实现首演。成功举办亚洲男篮锦标赛、湘江帆船赛、首届国际马拉松赛等活动。安全生产和食品药品安全监管不断加强，有效遏制了重大安全生产事故，保持重大食品药品安全事件“零记录”。实行信访首办责任制，依法治访、诉访分离有效落实。刑事发案率持续下降，群众安全感明显增强。社区矫正依法推进，法律援助不断拓展，社会矛盾纠纷有效化解。荣获首批全国和谐社区建设示范城市。

　　（七）着力政府自身建设。扎实开展“三严三实”专题教育，注重在项目建设、拆违控违等一线锤炼作风，展示了干部队伍攻坚克难、敢打硬仗的担当与作为。严格执行“三重一大”集体决策制，推行政府法律顾问制度，法治政府指数排名全国前列，荣获中国政府创新“最佳实践”奖。审计市本级政府投资项目885个，核减投资额36.2亿元。实施电子招投标试点。完成乡镇（街道）政务中心标准化建设，网上政府服务和电子监察全面实行。切实提升行政效能，发展环境明显优化。统计工作实现网格化数据质量控制。自觉接受人大和政协监督，认真落实人大及其常委会的决议决定，办理人大代表建议297件、政协委员提案387件。严格落实党风廉政建设主体责任和监督责任，风清气正的干事氛围更加浓厚。

　　密切军政军民团结，国防动员和后备力量建设不断加强，优抚安置等双拥共建工作取得新成果。

　　各民主党派、工商联、无党派人士及工会、共青团、妇联、科协、文联、侨联、红十字会、残联、社科联、贸促会、慈善会、关工委、老龄委等为长沙发展作出新贡献。国家安全、应急管理、民族宗教、防震减灾、人防、对台、档案、市志、机关事务管理、供销、海事、移民、气象、水文等工作取得新成绩。

　　各位代表，回首“十二五”，面对复杂多变的宏观形势，全市上下始终凝心聚力，精准发力，长沙发展的巨大成就令人鼓舞，站在新的起点乘势而上，我们充满自豪、更有信心！

　　——这五年，综合实力大步跨越，结构调整深入推进，为实现基本现代化奠定了坚实基础。经济总量位居全国省会城市第6位，人均地区生产总值超过11.6万元。财政总收入突破千亿大关。规模工业总产值迈入“万亿俱乐部”，规模工业增加值翻了一番多，高新技术产业增加值占地区生产总值比重达30.5%。培育形成新材料、工程机械、食品、电子信息、文化创意、旅游六大千亿产业集群，产业多点支撑格局初步形成。长沙县、浏阳市、宁乡县在全国县域经济发展中的排名大幅前移。

　　——这五年，城乡品质大幅提升，农业农村生机勃发，为推进城乡统筹发展探索了成功路径。望城撤县设区，城市建成区面积增加近一倍，“两桥两隧”、湘江综合枢纽等重大基础设施建成，构筑了“一江两岸，跨江发展”新格局。武广、沪昆高铁形成了“十”字交汇，黄花机场新航站楼竣工运营，磁浮工程实现机场与高铁站无缝连接，城市迈入地铁新时代，长湘、长浏、长韶娄等7条高速公路建成通车，现代立体交通体系日益完善。现代农业快速发展，城乡基础设施全面对接，农村“五大工程”稳步实施，基本公共服务惠及城乡，城镇化率达73.5%，富有特色的新型城镇、美丽乡村成为迷人的靓丽风景。

　　——这五年，资源节约成效显著，生态环境持续改善，为加速两型社会建设树立了长沙标杆。深入推进两型社会建设综合配套改革，大力推行节能、节地、节水、节材，创新生态补偿和排污权交易制度，国家节能减排财政政策综合试点全面完成，水、电、气阶梯价格改革取得明显成效，单位地区生产总值能耗累计下降20%以上，提前一年实现“十二五”节能减排目标。城乡环保和治理不断加强，新增造林绿化面积68.19万亩，主城区规模养殖全面退出。荣获联合国人居环境范例奖和中国可持续发展城市奖，获评全球绿色城市、全国十佳生态文明建设示范市，9个区县（市）全部成为国家生态示范区。

　　——这五年，社会事业全面发展，公共服务更加均等，为创造人民美好生活增添了幸福元素。城镇零就业家庭保持动态清零，大众创业、万众创新蔚然成风，城乡居民人均可支配收入年均分别增长11%、13%，人民生活更加殷实。企业退休人员养老待遇“十一连增”，城乡居民养老、医保统筹一体化和大病保险实现全覆盖，社会保障不断完善。率先普及十二年教育，全面建成国家义务教育基本均衡市。成为首批国家公共文化服务示范区，荣获全国平安建设“长安杯”，蝉联全国文明城市，连续8年获评最具幸福感城市。

　　回顾“十二五”发展，我们深刻体会到：必须致力科学发展，尊重经济规律，注重发展质量和效益，转变发展方式，优化经济结构，改善生态环境，心无旁骛办好长沙的事情；必须坚持改革创新，解放思想、求新求变，在抢抓机遇中走在前列，形成引领发展、率先发展的强大势能；必须树立底线思维，以问题为导向，以民生为根本，努力使群众的获得感与发展的速度质量同步提升；必须强化作风保障，围绕市委的战略目标，坚定方向、凝聚人心、善于攻坚、勇于担当，切实形成团结奋进的发展局面。

　　各位代表，过去五年的成绩，是省委、省政府和市委正确领导的结果，得益于市人大、市政协的监督和支持，凝聚了全市人民的辛勤与智慧。在此，我代表市人民政府，向全市各族人民，向各民主党派、工商联、无党派人士、人民团体，向驻长部队、武警官兵和政法干警，向国内外所有关心支持长沙发展的各界朋友，表示诚挚的感谢和崇高的敬意！

　　在总结成绩的同时，我们也清醒地看到发展中的问题和不足：产业转型创新发展瓶颈依然突出，发展动能亟待转换；企业成本上升、利润下降，发展还面临不少困难；外贸规模不大、拉动力不强，开放型经济短板有待补齐；发展深层次矛盾日益显现，改革难度更大；城市发展新旧问题交织，规划建设管理需要进一步创新；环境承载能力不断下降，水和空气治理尤需加大力度；群众出行、教育、医疗、就业等难题仍待破解，改善民生还须付出更大努力；少数干部服务企业和群众的主动性不够，政府服务质量和办事效率与群众期待还有差距；影响社会和谐稳定的矛盾和问题仍然较多，安全生产基础还不牢固，社会风险防控和应急处置能力还需增强等。我们将直面问题，迎难而上，采取有效措施，切实加以解决。

　　二、“十三五”时期目标与任务

　　“十三五”时期，我国经济长期向好的基本面没有变。长沙既具备加快发展的有利条件，也面临转型创新发展的艰巨任务，仍处于大有作为的重要战略机遇期。“十三五”是全面小康向基本现代化迈进的攻坚期。按照市委确定的2017年率先建成全面小康、2022年实现基本现代化阶段性目标的战略部署，长沙面临双重发展任务和诸多风险挑战，既要破解难题，又要引领发展，必须以更大的决心和勇气攻坚克难，不断开拓发展新境界。“十三五”是产业转型升级的关键期。传统产业比较优势需要巩固，新兴产业培育壮大尚待时日，产业结构缺乏竞争力，只有抓住机遇，补齐短板，促进产业加快迈向中高端，才能牢牢把握发展主动权。“十三五”是新旧发展动力的转换期。新常态下，旧的发展动力逐步减弱，过于依赖要素投入的增长模式难以持续，迫切需要将发展动力转换到创新驱动上来，用发展新动力开拓发展新空间。

　　“十三五”时期，认识新常态、适应新常态、引领新常态，必须紧紧围绕“率先建成全面小康、加快实现基本现代化”的总目标，始终坚持创新、协调、绿色、开放、共享的发展理念，挖掘增长潜力，厚植发展优势，努力建设能量更大、实力更强、城乡更美、民生更爽的长沙。

　　未来五年主要预期目标：地区生产总值年均增长9%；规模工业增加值年均增长9%；固定资产投资年均增长12%；社会消费品零售总额年均增长10.5%；进出口总额年均增长10%；财政总收入年均增长10%；城镇登记失业率控制在4%以内；城乡居民人均可支配收入年均增长9%；价格总水平基本稳定；单位地区生产总值能耗、二氧化碳和主要污染物排放总量达到省定目标。重点抓好五大任务：

　　（一）推进开放融合，更加凸显国家战略支点城市地位。深度融入大格局。主动对接国家战略，积极建设“一带一路”重要节点城市、长江经济带中心城市，进一步增强长沙的战略影响力和发展辐射力。全力培育“双引擎”。围绕“三区一高地”战略定位，发挥湖南湘江新区的引领作用，充分调动相关区县、园区积极性，加快构建“一区带多园”的融合发展格局，努力建设新的核心增长极；以空港新城、高铁新城、长沙港、金阳新区等为支点，加快建设东部开放型经济走廊，实现“东西比翼、双核驱动”发展。精准施策“补短板”。加快补齐现代服务业、开放型经济短板，大力发展跨境电子商务，促进服务业转型升级；推动外贸向优质优价、优进优出转变，构筑内陆开放型经济新高地。

　　（二）推进创新驱动，全面激发创新创业引领城市活力。打造中部创新中心。全面建设国家自主创新示范区，充分发挥人才、企业的主体作用和园区的载体作用，实施产业关键技术攻关工程，力争建设100个市级以上示范众创、众包、众扶、众筹、众智平台。建设智能制造强市。引导产业提质转型，大力发展先进制造业，实施“制造+互联网+服务”工程，推动制造业向智能化、高端化、低碳化升级，构建现代智造体系，打造具有国际竞争力的智能制造基地。优化创新创业生态。完善普惠性创新支持政策，降低创新创业门槛，促进科技创新与大众创业深度融合，弘扬创新创业文化，让创新创业者成为真正的时代英雄。

　　（三）推进城乡统筹，精心擦亮两型宜居品质城市名片。创造优良人居环境。统筹生产、生活、生态布局，增强城市内部布局的合理性，提升城市的通透性和微循环能力。控制城市开发强度，着力整治大气、水体、土壤等污染，科学解决垃圾处理问题，连通江河湖库水系，编织城乡绿网，增加城乡绿量，建设全国水生态文明城市和国家生态园林城市。统筹规划建设管理。树立“精明增长”、“紧凑城市”理念，加强对城市空间立体性、平面协调性、风貌整体性、文脉延续性的规划管控，科学谋划城市“成长坐标”。加强地上地下基础设施建设，完善城市公共配套服务能力。建设空港综合交通枢纽，构建“米”字形轨道交通格局，完善城市快速道路网络，不断增强城市综合承载能力。创新城市治理方式，加强城市精细化管理，让市民在城市生活得更方便、更舒心、更美好。促进城乡融合互动。深入推进国家新型城镇化综合试点，加快农业转移人口市民化，促进中心城区与次中心、小城市、中心镇、特色镇协调共进发展。加强城乡基础设施对接，实施新一轮农村公路、电网、宽带、自来水工程，建设美丽乡村，让农村居民与城市市民同享幸福生活。

　　（四）推进文化引领，切实彰显湖湘文化标识城市特质。传承城市历史文脉。加强历史文化遗存和非物质文化遗产保护，让古城、古镇、古物等传承长沙记忆，让历史文化与现代文明交相辉映。推动文化跨界融合。深度发掘湖湘文化底蕴，加强文化与资本对接，打造具有全国影响力的文化产业集群，提升文化竞争力。积极促进文化对外交流，让湖湘文化走向世界，让“文化+”增添城市新活力。健全文化服务体系。加强文化基础设施建设，丰富精神文化产品，创新文化供给方式，满足群众公共文化需求，提升人文素养，塑造国际文化名城精神风貌。

　　（五）推进民生改善，全面共享和谐幸福典范城市成果。守住民生底线。切实抓好就业、社保、养老、住房等基本民生保障，实现应保尽保。率先全省打赢脱贫攻坚战，2017年全面完成贫困村“摘帽”、建档立卡贫困户清零任务，坚决不让困难地区和困难群众在全面小康中掉队。繁荣社会事业。创建儿童友好型城市和学习型城市，促进各类教育协调发展。扩大优质中小学覆盖面，实现就近上好学。优化城乡卫生资源配置，保障食品药品安全，加快体育事业发展，基本建成健康长沙。创新社会治理。拓宽社情民意表达渠道，强化社会矛盾排查处理，维护人民生命财产安全，实现政府治理、社会调节和居民自治良性互动，把长沙建设成为最具安全感、公平感、正义感的城市。

　　实现上述目标和任务，在工作中必须牢牢把握以下几点：

　　一是更加注重结构性改革。顺应技术、人口、消费习惯的新变化，把准产业脉搏，在供需两端发力的同时，更加注重供给侧结构性改革，积极培育新的发展动能。

　　二是更加注重融合发展。把握时代发展大势，加快推进产业融合、产城融合、区域融合、城乡融合，努力走出一条以融合发展放大自身优势、拓展发展空间的新路子。

　　三是更加注重市场力量。充分发挥市场在资源配置中的决定性作用，扩大政府和社会资本合作领域，让更多企业借力新一轮资本市场改革做大做强。

　　四是更加注重服务企业。搭建企业综合服务平台，帮助企业破解发展难题，优化营商环境，激发企业家精神，为企业发展提供更加适宜的土壤、更加滋润的雨露、更加灿烂的阳光。

　　五是更加注重法治思维。以法治政府引领法治城市、法治社会建设，让循法治、讲道德、重诚信、守契约成为风尚，让所有市场主体和人民群众的正当权益依法得到保障。

　　六是更加注重共建共享。始终坚持民生优先，让群众得到更多实惠。充分调动全市人民特别是企业家、创新人才的积极性、创造性，让每一位“长沙人”都有干事的舞台、出彩的机会。

　　三、2016年工作重点

　　2016年是实施“十三五”规划元年，政府工作的总体要求是：全面贯彻党的十八大和十八届三中、四中、五中全会精神，以邓小平理论、“三个代表”重要思想、科学发展观为指导，深入贯彻习近平总书记系列重要讲话精神，认真落实中央经济工作、中央城市工作会议和省、市委经济工作会议部署，紧紧围绕率先建成全面小康、加快实现基本现代化的总目标，以“四个全面”战略布局为统领，贯彻“五个发展”新理念，继续坚持“稳住、进好、调优”总要求，在推进“三量齐升”、“五化同步”中走在前列，奋力实现“十三五”发展精彩开局。

　　全市经济社会发展的主要预期目标是：地区生产总值增长9.5%左右；规模工业增加值增长9.5%；社会消费品零售总额增长12%；固定资产投资增长15%；财政总收入增长10%；城乡居民人均可支配收入分别增长9%。突出抓好八个方面的工作：

　　（一）突出重大项目建设。发挥产业项目支撑作用。力促比亚迪电动卡车及特种车、湖南粮食集团宁乡产业园、网讯通、梅溪湖国际文化艺术中心等项目建成，加快推进联通长沙云数据中心、格力电器、湘江涂料、华谊影城等项目建设。提升基础设施承载功能。启动地铁6号线建设，加快3、4、5号线建设，全面铺开火车货运新北站二期建设。推进湘府路、湘江大道、三一大道快速化改造，加快南北横线、金阳大道、机场联络线、中青路和车站路北延线等道路和蒙华铁路建设，实现大围山旅游干线通车，启用火车南站东广场，加快汽车南站综合枢纽建设。强化项目建设要素保障。推动融资平台转型发展，充分发挥产业投资基金的撬动作用，积极与各类金融机构合作，吸引更多社会资本参与基础设施、产业发展和民生项目建设。推进拆迁腾地、闲置土地清理，切实强化用地保障。

　　（二）突出产业结构调整。加快发展先进制造业。以智能制造为统领，以重大技改项目为抓手，引导威胜、博世等企业实施产品和生产过程智能化改造，建设工业云平台，举办智能制造大会，推动制造业向中高端发展。巩固提升工程机械、花炮等传统产业，调整优化汽车、新材料、电子信息、食品加工等支柱产业，培育壮大工业机器人、增材制造等先进制造业。大力发展新兴产业。实施“互联网+”行动，加强大数据开发与应用，培育壮大新产业、新业态。加快58众创等一批示范性创客空间建设，拓展生活类移动互联网应用，鼓励发展创新创意产业，以新产品创造新供给。推进装配式建筑、新能源汽车、生物医药、文化创意、节能环保等新兴产业发展，抓好北斗导航、苏宁云商等重大项目建设。积极发展现代服务业。释放消费潜力，扩大消费需求。支持现代金融、现代物流、服务外包、设计咨询、商务会展、检验检测等生产性服务业发展，建设湘江西岸现代服务业走廊，推进浏阳河文化旅游产业带、湖南地理信息产业园、后湖文化艺术产业园、滨江金融商务区建设。建成长沙国际会展中心，首展举办住宅产业博览会。组建长沙农村商业银行，积极支持长沙银行上市，规划建设金融创新特色产业园。提质改造岳麓山景区，支持创建大围山5A级景区，加快以铜官旅游度假区为重点的湘江古镇群建设，打造“智慧旅游”城市。增强园区发展活力。突出工业园区的主战场地位，引导高端产业、优质项目、知名企业在园区合理布局，推进园区差异化、特色化、专业化发展。优化管理体制、运行机制，赋予园区更大自主权限。创新园区绩效考核指标体系，突出园区发展特色，提高单位面积产出，增强园区竞争力。

　　（三）突出战略平台带动。全面提速湖南湘江新区建设。围绕新区发展战略定位，进一步完善顶层设计和专项规划，深化要素供给、行政审批、生态文明等领域体制改革，引进一批重大战略投资者，加快培育高端产业，强化统筹管理，发挥国家级新区的示范带动作用，合力打造长沙发展“新引擎”。全面推进国家自主创新示范区建设。实施《自主创新示范区建设中长期规划》，全面创新科技管理、科技评价等体制机制。发挥工业机器人、增材制造等工业研究院孵化作用，推进湖南省大学科技产业园等创新平台建设。加强知识产权保护，支持具有自主知识产权的产品首购。促进金融创新与技术创新深度融合，让资本跟着技术走、技术跟着市场走，加速科技成果产权化、知识产权产业化。推进军民融合创新示范，鼓励优势企业参与军品研制生产。继续推进“3635”行动计划，引进和培育紧缺型战略创新人才与团队。全面深化两型社会建设。严格环保执法，探索实施土地开发利用强度和效益考核。启动区域能源中心、再生水厂等一批示范项目，加快浏阳两型产业园建设。开展“绿色城市”四大工程建设，抓好绿心保护、磁浮两厢生态景观和环城绿带生态圈建设，新增城市绿地600公顷。加快新能源汽车推广及配套充电桩建设，淘汰2005年以前注册的营运黄标车。推进海绵城市建设。加强湘江长沙段流域综合治理。全面推进建成区黑臭水体治理。全面加快临空经济示范区建设。高起点规划建设空港新城，高品质建设发展高铁新城，加速黄花机场二跑道建设，推动空港、高铁有效对接，加快筹建黄花综合保税区，构建东部开放型经济走廊的强力支撑。

　　（四）突出加强城市工作。优化城市空间布局。实施“多规合一”，加强滨水、临空、交通枢纽、历史街区等城市节点设计，完善跳马、暮云等区域规划，整合城市基础地理信息数据平台。加快黎托新城、省府新区、望城滨江、宁乡沩东等片区建设，推进湘江西岸提质改造，打造新城新形象。完善城市综合功能。改造德雅路、杨家山立交桥，推进含浦大道、枫林路三期等道路建设，加快曙光南路、洞井路等城市主干道跨京广铁路桥建设，打造示范精品干道10条，改造背街小巷100条。完成桥梁绿化亮化提质50座。建设公共停车场120个、新增车位2.6万个。坚持公交优先战略，开展“公交都市建设年”活动，新增公交专用道100公里，改造港湾式公交中途站100个，优化公交网线30条，实现公交车全部进站场；推广公共自行车租赁，打造慢行系统；加强公交管理，提供优质服务，深化出租车行业改革，让公共交通成为市民出行首选。加强新城区地下综合管廊建设，新建梅溪湖等地下综合管廊7条。启动敢胜垸、苏家圫、雷锋、星沙北污水处理厂建设。提质改造南郊公园，加快鹅羊山、长沙儿童公园建设，启动狮子山、石人冲公园建设。加快片区提质改造。重点推动朝正垸、老火车北站、黄兴北路、雅塘等片区改造。加大棚改攻坚力度，完成长沙机床厂、白沙液街、南门口、三汊矶等棚改3.3万户。提质改造老旧社区50个，完成新桥村等城中村改造。加快推进安居工程建设，促进建筑业转型，加快房地产去库存，引导房地产市场稳定健康发展。提升城市管理水平。坚持城管重心下移、力量下沉，发挥基层一线和市民在城市管理中的主体作用，以机械化、智能化手段提升城市管理效率。改进城管考核机制，将考核从街道向社区延伸。加强户外广告管理和电子显示屏整治，继续深化拆违控违行动。充分发挥物业维修基金作用，重点加强小区老旧电梯安全管护。提升市民文明素养，建设更高水平的文明城市。

　　（五）突出城乡协调发展。大力发展现代农业。加快重大现代农业项目建设，引导发展高效生态农业。培育新型农业经营主体、新型职业农民，推进农业标准化、品牌化建设，引导社会资本投入农业领域，激发农业经济发展活力。巩固农业基础地位，健全农业投入体制机制，抓好“米袋子”、“菜篮子”工程建设，完善农村物流配送体系，确保主要农产品稳定安全供应。推进花卉苗木产业升级，打造百亿产业集群，提升浏阳黑山羊、宁乡花猪产业化水平。深入开展国家新型城镇化综合试点。探索城镇化投融资新模式，重点抓好小城镇建设。着力打造美丽乡村，促进镇村联动发展。改造农村危房5000户、危桥15座，实施普通公路安保工程100公里，农村自来水普及率提高到70%以上。持续推进农村环境综合整治，完成无害化厕所建设1万座。加强农村特色民居保护，大力发展乡村旅游。切实优化农村教育、医疗、养老等公共服务，让群众在家门口分享城镇化成果。创新城乡统筹发展机制。实施农村土地“三权分置”和农村宅基地改革试点，加快农村土地确权登记颁证工作，推广土地合作经营新方式。全面完成并村工作。推动“互联网+”向农村延伸，促进城乡基础设施和公共服务有效对接。深入推进长沙现代农业综合配套改革试验区建设，以制度创新激发农业农村发展潜力。

　　（六）突出全面深化改革。扎实推进经济领域改革。基本完成新一轮国有企业战略重组，实现政企、事企完全脱钩、移交，建立以管资本为主的国资监管制度体系，完成国企负责人薪酬制度改革。细化全口径预算，抓好乡镇国库集中支付改革。深化供销合作社综合改革。深入推进行政体制改革。清理规范行政审批中介服务，完成园区行政审批制度改革。试点证照分离制度，推行企业集群注册，构建全市统一的商事登记监管平台。完善城市综合执法体制。探索农业领域综合执法。改革机关事业单位养老保险制度。有序推进社会领域改革。完成户籍制度改革和不动产统一登记基础工作。加大公立医院改革力度，鼓励社会资本举办医疗机构。以文化社会化改革为导向，扶持民间文化机构发展。创新体育服务供给，促进群众体育、竞技体育与体育产业全面协调发展。大力发展社会组织，支持社会组织参与社会管理和公共服务。加快推进生态文明体制改革。推行两型认证制度，建立绿色发展评估指数体系，健全环境损害赔偿制度。开展城区生活垃圾分类收集处理试点，实现主城区餐厨垃圾收集处理全覆盖。

　　（七）突出推进双向开放。坚持内外贸并举。加快申报跨境电商进口试点城市和金霞经开区铁路一级口岸，打造外贸综合服务、通关代理中心等外贸服务载体；支持各类贸易主体发展，推动市场采购贸易试点；落实专业市场规划，继续推进专业市场迁建和提质改造，支持高岭、金桥、湾田、大河西农产品物流中心等市场发展；科学规划现代仓储物流业发展。扩大对外开放。开拓国际合作渠道，开通至澳洲、俄罗斯、日本等国际航线和货运包机航班；支持优势企业“抱团出海”，鼓励工程机械、文化创意等产业组成“产业联盟”参与全球竞争，建设境外经济贸易合作园区。推进精准招商。围绕产业链招大引强，在产业承接、产业延伸上精选项目，着力引进世界500强、央企和国内知名企业；注重招商实效，提高签约项目的履约率、资金到位率和开工率。

　　（八）突出增进人民福祉。切实办好民生实事。完成自来水户表改造10万户；建设32个乡镇（街道）户外全民健身中心和1000处全民健身场所；建设示范性乡镇（街道）综合文化站30个，开展“好戏天天送”700场，实施农村广播“村村响”工程；改扩建敬老院14所，建设街道老年人日间照料中心24家；建设能源环保网络服务站25个；创建绿色示范村、社区各40个；基本解决城区房产办证遗留问题。全面推进脱贫攻坚。坚持分类管理，因户施策，精准把握群众需求，精准扶持产业发展，精准改善基础条件，减少贫困人口5万人以上。增加公共服务供给。推行政府购买服务，多渠道满足公共服务需求。促进更高质量的就业，城镇新增就业12万人，扶持4000名大学生创业。完成全民参保登记，扩大社会保险征缴面。推进长沙县、望城区城乡低保一体化。创建义务教育标准化学校80所，建设公办幼儿园30所；推行“阳光招生”，逐步化解大班额问题；实施卓越教师培养工程；支持长沙商贸旅游职院等创建省卓越职业院校，整体迁建长沙卫生职院。实施全面两孩政策。深化提升基层医疗卫生服务三年行动，推行城乡居民大病保险。加强区县社会福利中心建设，全面深化养老服务业综合试点，居家养老服务设施加快覆盖农村社区。不断完善现代体育公共服务体系，市全民体育健身中心全面开工。推进长沙国王陵、炭河里等考古公园和潮宗街、坡子街等历史文化街区建设，加强文化遗产保护利用，提质改造非遗展示馆和美术馆，留住城市历史记忆。加强文化市场监管服务，净化社会文化环境。着力建设平安长沙。强化法治宣传教育，完善社会治安立体防控体系，提升警务信息化水平，加强城郊警务站建设，强力推进禁毒工作综合治理，重拳打击各类违法犯罪行为。积极办理法律援助案件，建设青少年法治教育中心和9个公共法律服务中心。强化源头治访、依法治访，着力化解信访积案。完善排查调处机制，有效化解社会矛盾纠纷。加强宗教场所管理，维护民族团结和谐。理顺社区职能，提升服务能力。严格落实安全生产责任，严防重特大安全事故发生。积极创建国家食品安全城市，完成肉菜流通追溯体系一期建设，建好100个农贸市场食品安全快检室，让人民群众吃得放心。

　　支持国防建设和军队改革，推进军民融合深度发展，提升国防动员能力，做好双拥共建、优抚安置和人民防空等工作，争创全国双拥模范城“六连冠”。

　　与此同时，实施质量强市战略，提升产品、工程、环境和服务质量。精心组织第三次农业普查。保障妇女、儿童、老年人、残疾人合法权益，支持工会、共青团、工商联、科协、文联、侨联、红十字会、社科联、贸促会、慈善会等群团组织工作。全面加强国家安全、应急管理、防震减灾、对台、档案、市志、机关事务、海事、移民、气象、水文等工作。

　　各位代表，实现“十三五”发展目标，完成今年各项任务，必须切实改进政府工作，努力建设人民满意的政府。

　　建设法治政府。完善重大行政决策机制，健全法治政府建设指标体系，推进机构、职能、权限、程序、责任法定化。坚持依法用权，理顺行政执法体制，加大行政问责力度，提高运用法治思维和法治方式的能力。自觉接受市人大及其常委会的法律监督、工作监督和市政协的民主监督，定期向人大报告工作、向政协通报情况，认真办理人大代表建议和政协委员提案。

　　建设效能政府。厘清行政权力边界，整合行政资源，切实转变政府职能。深化政务公开，及时回应社会关切，把政府工作置于群众监督之下。启动“政务云”建设，破除政府部门信息“孤岛”，让数据多跑路、群众少跑腿，打造智慧型政府，努力建设审批环节少、办事效率高、服务质量好的城市。

　　建设务实政府。切实改进工作作风，提升政府执行力。高度重视群众切身感受，主动深入问题多、困难大的地方听民声、帮民困、解民忧。坚持关注基层、重视基层，设身处地解决基层发展难题，为基层一线营造更优的工作环境。旗帜鲜明地支持改革创新，让实干成为政府的作风导向，让担当成为干部的自觉追求。

　　建设廉洁政府。政府财力都来自于人民，必须厉行节约、反对浪费，每一分钱都要用在“刀刃”上。强化效能监察和绩效管理。建成统一开放的公共资源交易市场。加强政府合同审查与管理。稳步推进公务用车制度改革。延伸经济责任审计领域，强化对重点领域、关键环节、重要岗位权力运行的监督制约。坚持讲规矩、守纪律，努力建设一支人民满意的公务员队伍。

　　各位代表，立足新起点，展望新征程，我们深感使命重大，责任在肩。让我们在省委、省政府和市委的正确领导下，紧紧团结和依靠全市人民，夙夜在公，勤勉奋进，用激情绘就蓝图，用实干成就梦想，努力建设能量更大、实力更强、城乡更美、民生更爽的长沙！
